# Proper positioning of mice for Cobb angle radiographic measurements

**DOI:** 10.1186/s12891-021-03949-8

**Published:** 2021-01-12

**Authors:** Zhe Yi Chen, Keith Dip Kei Luk, You Qiang Song, Bo Gao, Jason Pui Yin Cheung

**Affiliations:** 1 Department of Orthopaedics and Traumatology, The University of Hong Kong, Professorial Block, 5th Floor, 102 Pokfulam Road, Pokfulam, Hong Kong SAR, China; 2 School of Biomedical Sciences, The University of Hong Kong, Pokfulam, Hong Kong SAR, China

**Keywords:** Scoliosis, Mouse, Radiograph, Cobb angle, Positioning

## Abstract

**Background:**

There is no recommended standard for positioning of a mouse for radiographic assessment of the spine. This is necessary to have reproducible radiographic data and avoid false positive results. The objective of this study was to investigate the impact of various postures on Cobb angle measurements and to set up a positioning standard for imaging mouse spines.

**Methods:**

This study was conducted in three parts. Firstly, we identified the problem of lack of posture standardization for radiographs. We collected 77 C57BL/6 J mice for spine radiographs and found a scoliosis prevalence of 28.6% with large variations in curve magnitude. Secondly, 24 C57BL/6 J mice underwent 4 consecutive weekly radiographs and observed high variations (relative standard deviation: 125.3%) between radiographs. Thirdly, we collected another 82 C57BL/6 J mice and designed 14 different postures that could take place during imaging. These postures were related to curling of the limbs, and head, pelvic and tail tilting.

**Results:**

The results showed that head and pelvic tilting significantly affects the curve magnitude with effect size (Glass’s delta) over 1.50. Avoiding these incorrect positions during radiographs is warranted. The standard recommended posture for mouse imaging entails positioning the snout, interorbital space, neck and whole spine in one line, and with the limbs placed symmetrical to the trunk, whilst avoiding stretching the body of the mouse.

**Conclusions:**

Our work exemplified the importance of standard protocol during imaging when using an animal model in the scoliosis study. We recommend utilizing this standard in studying various disorders of the spine to avoid technical causes for the appearance of a curve.

## Background

Scoliosis is defined by Cobb angle measurement greater than 10° from a posteroanterior standing radiograph [1]. The accuracy of measurement is crucial as different angle ranges indicate different treatment options [2–4]. For humans, the effect of posture on the outcome of radiological measurements is well-established. A research conducted by Ramirez et al [5] showed that the mean difference in Cobb angle ranges from 6° to 12° between sitting and other positions. Simply lying supine may produce significant changes in the curve magnitude due to spinal flexibility [6, 7]. A cut-off of 5° is often used to identify significant changes in the curve [8].

Scoliosis animal models are necessary for establishing and testing mechanical and genetic etiologies. Quadrupedal animal models include pigs [9], dogs [10], rabbits [11], calves [12], goats [13] and rats [14], while bipedal animal models include chicken [15], non-human primates [16] and rodents [17]. Bipedals have curves influenced by gravity which is like the situation in humans while quadrupedal animals’ curves may be more physiological and is affected by muscle tone and posture. The mouse in particular is often used as it is easy for genetic manipulation and also suitable for mechanical testing. As we begin to utilize animal models to study scoliosis, there is a need to produce the same guidelines for animal positioning during radiographs. However, there are no such standardized recommendations produced. Scoliosis is a 3-dimensional deformity and the degree of rotation may lead to variations in measuring its angle on an anteroposterior radiograph [18]. A standardized approach to obtaining these radiographs is necessary to allow for consistency in communication and accuracy in data reporting. Hence, we aim to study the effects of various postures on Cobb angle measurements in mice and to provide a feasible standard of positioning that constrains posture related variations in measurement.

## Methods

This was a three-part radiographic study. The first part was used to identify the problem with unstandardized posture for radiographs, the second was to identify variations between multiple radiographs, and the third part was to study the effects with varied malpositioning. We utilized C57BL/6 J mice for this study. For sample size of animal tests, we aim to utilize at least 12 mice per experiment, following a protocol on a previous interventional mouse model [19]. Based on the numbers of mice in the three experiments as seen below, our sample size was adequate for such study. All mice were anesthetized by intraperitoneal injection of 5 mg/ml phenobarbital sodium (Dorminal 20%, Alfamedic) dissolved in saline at the dose of 50 mg/kg. After losing reflection to firm pinch on the paw, they were immediately transferred to warm pad for imaging. All radiographs were completed within an hour to guarantee the mice in the best stationary state. After the experiments, they were put back to the warm pad until sober. Weight and feeding behaviors were monitored for several subsequent days. All anesthetic and radiographic studies were performed by the same investigator. All animal experiments were approved by the Committee on the Use of Live Animals in Teaching and Research (CULATR) (Ref# 3720–15).

### Cobb angle measurements

**Fig. 1:**
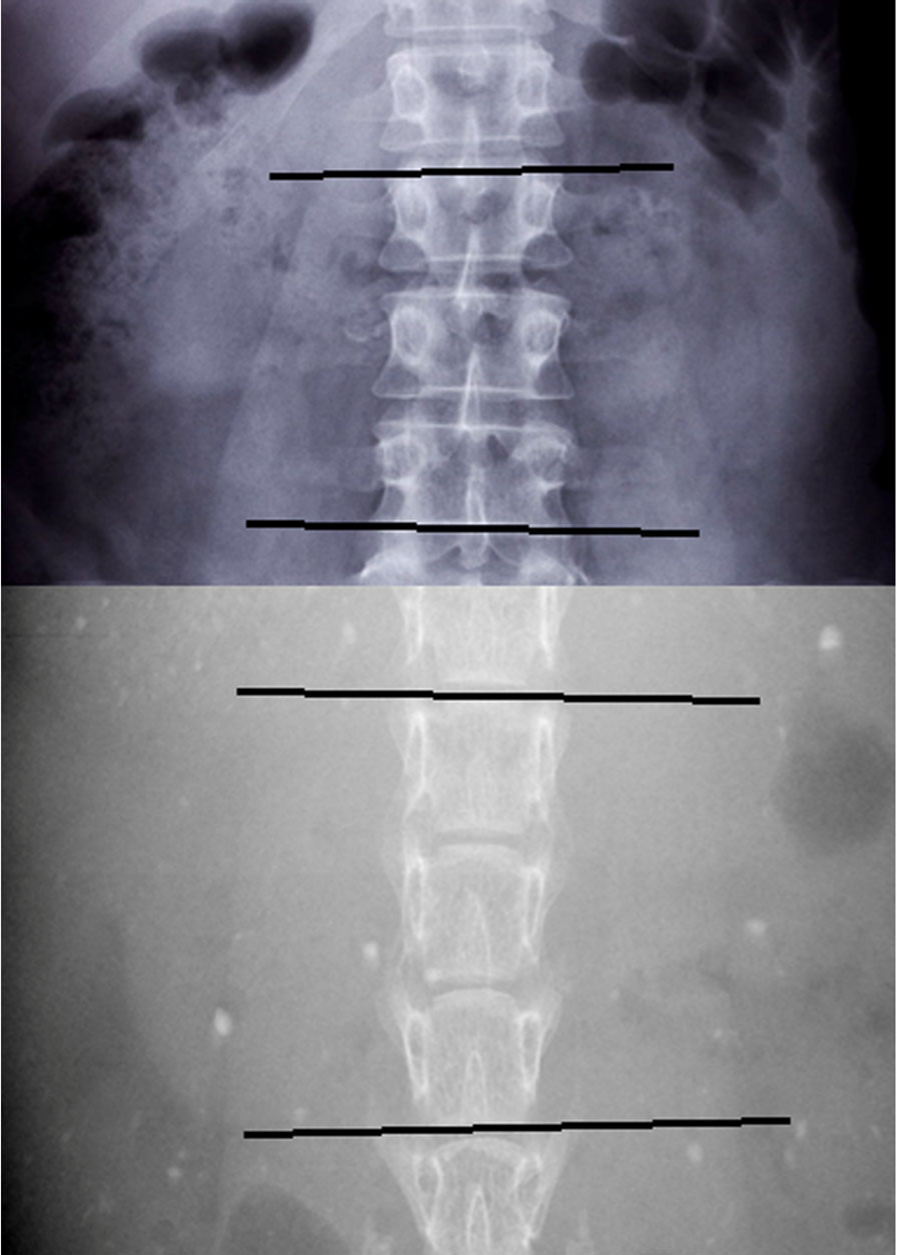
Comparison of a human (top) and mouse (bottom) vertebrae. The upper and lower endplates are easily seen in the mouse vertebrae and are indicated by the black lines

All radiographic Cobb angle measurements were performed in AUTOCAD (version 0.48.M.570;© 2017 Autodesk, Inc.). Tiff image files were imported by Raster image reference. The measurement method used was the same as in humans with an angle formed by the superior endplate of the most tilted cranial vertebra from the apex and the inferior endplate of the most tilted caudal vertebra from the apex (Fig. 1). For the mouse, the upper and lower endplates of each vertebra can also be seen clearly for the measurement. Measurements were carried out with the reader blinded to the mouse number, and each image was measured twice with an interval of one month. All measurements were within 5° and the final reading for analysis was the average of the two measurements.

### Preliminary experiment

**Fig. 2:**
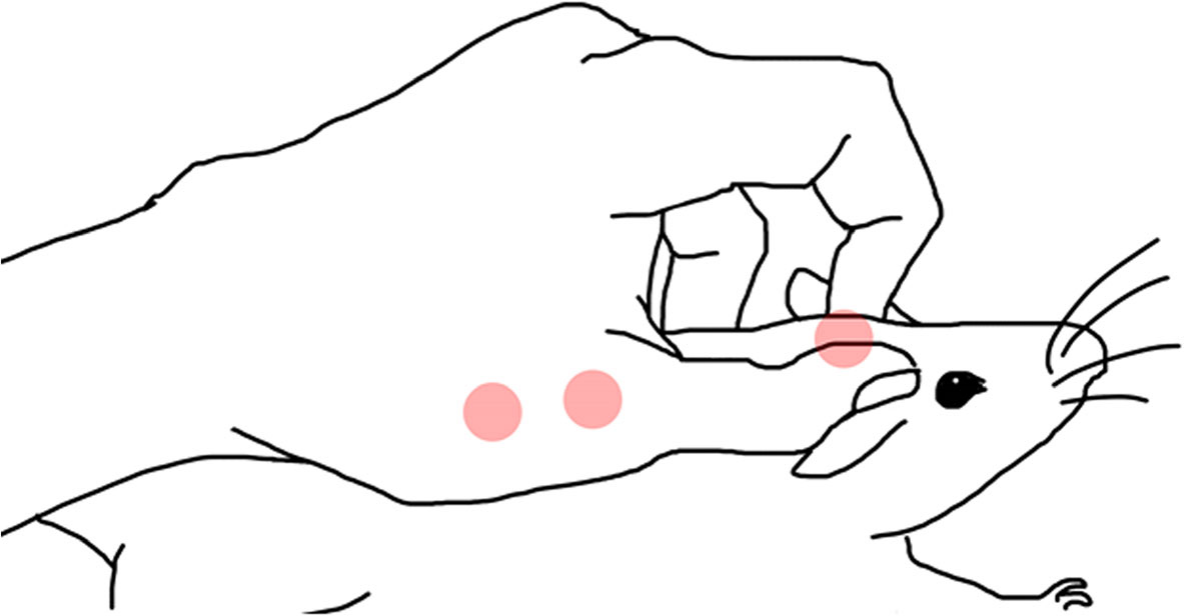
Illustration of how to hold and move the mouse. The three holding points are highlighted with red dots

**Fig. 3:**
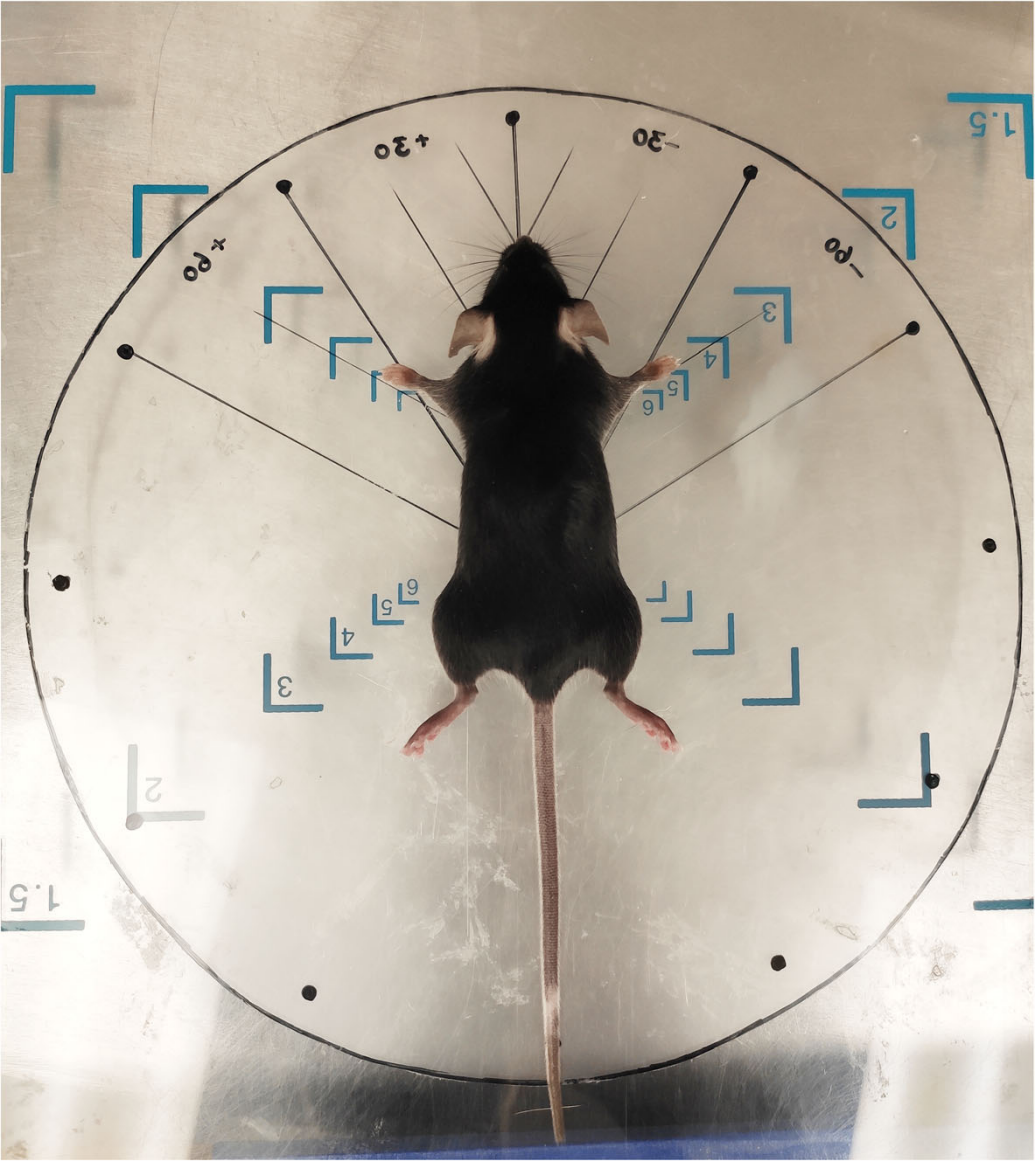
Illustration of the plastic pad used for postural angle quantification, and the “correct posture”

For the first stage of the radiographic study, we collected 77 wild-type C57BL/6 J mice with no documented genetic susceptibility to spinal deformities to perform radiographs. There were 39 females and 38 males with age (mean ± standard deviation/SD) of 166 ± 68 days. This was a general litter available to our faculty. A digital diagnostic X-ray machine (KeenRay Top-U) was used to obtain plain radiographs at 40 kilovoltage (kv) and 40 mA (mAs). After anesthesia, mice were kept in the prone position until they were transferred to the examining table right beneath the radiation source. During transfer, we used the index finger and the thumb to clamp the scruff in addition to pinching the back fur between the middle finger/fourth finger and the thenar eminence (Fig. 2). This formed three holding points to allow horizontal movement while maintaining the natural status of the spine. Because the mouse was biologically left-right symmetrical, after it was put on the table, we adjusted the head and limbs by gentle movements to make sure that the snout, interorbital space, cervical vertebrae, spine and caudal vertebrae were kept in a line, and the limbs were placed on the board naturally while symmetrical to the trunk. This posture was defined as the “correct posture” (Fig. 3). No outer force was applied to the spine. A position and reposition test was done to confirm repeatability of the positioning maneuver before proceeding with the x-rays.

Direction of curves was not taken into account for this part of the study and so all angles were considered positive. Descriptive Statistics for mean, SD and standard error (SE) of mean calculations were used to describe the data. All statistical analyses were performed using SPSS IBM Statistics 20 (IBM, Armonk, New York, USA). A *p* value of < 0.05 was considered statistically significant.

### Weekly X-ray experiments

For the second study, we explored the variation of curve magnitude measurements with multiple radiographs. We collected 24 wild-type C57BL/6 J mice with no documented genetic susceptibility to spinal deformities for 4 consecutive weekly X-rays. There were 5 females and 19 males. The first radiograph was taken at the age of 4 weeks. We followed the standard rule and used the correct posture described above for radiographs. Relative standard deviation (RSD) was introduced for normalization, and SDs for RSD of the 24 subjects were calculated and used to describe the overall variation in multiple measurements. All calculations were reported by the descriptive statistics.

### Position study

For this third part, we designed 14 different postures to test the impact of each malpositioning on the spinal curvature. These postures were based on the three main movable parts of mouse namely the limbs, head and tail. The postures are as follows:

1. 4 postures related to the curling of limbs (Curled left forelimb, Curled right forelimb, Curled left hindlimb and Curled right hindlimb).
2. 4 postures related to head tilt (Head tilt left 30°, Head tilt left 60°, Head tilt right 30° and Head tilt right 60°). The angle was formed by the longitudinal axis of the trunk and the axis of the head.
3. 2 postures related to pelvic tilt (Pelvis tilt left 30° and Pelvis tilt right 30°). The angle was formed by the longitudinal axis of the trunk and the top of the pelvis.
4. 2 postures related to tail tilting (Tail tilt left 30° and Tail tilt right 30°).
5. 2 postures related to head rotation (Head rotates clockwise and Head rotates anti-clockwise) in the direction of the observer.

For this part of the study, 82 wild-type C57BL/6 J mice with no documented genetic susceptibility to spinal deformities were collected. There were 37 females and 45 males. The mean age was 54.5 ± 1.7 days. Each mouse was applied to each of the 14 postures as well as the “correct posture”. In order to quantify the tilt angles, a round plastic pad was made which was 20 cm in diameter and placed over the imaging platform. It was labeled every 30° with branch-like auxiliary lines for accurate malpositioning of the mouse (Fig. 3). ULTRAFOCUS (Faxitron Bioptics LLC) was used to obtain plain radiographs at 25 kv, 3.00s and 0.4 mA. After anesthesia, mice were kept in the prone position until they were transferred to the tray with the method described before.

**Fig. 4:**
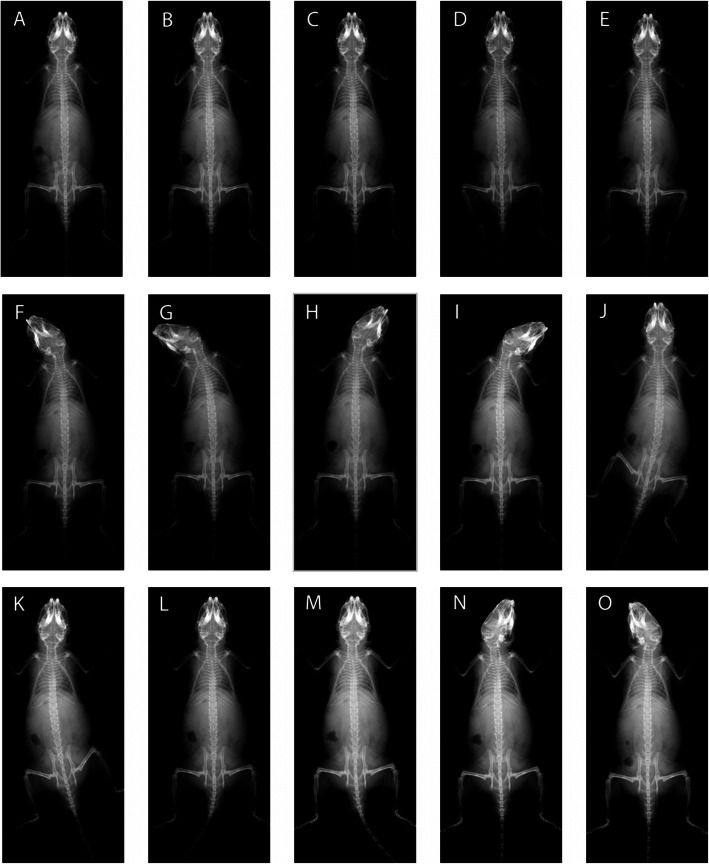
Radiographs of the same mouse with 14 different postures (**b**-**o**) plus one “correct posture” (**a**).; (**b**) Curled left forelimb; (**c**) Curled right forelimb; (**d**) Curled left hindlimb; (**e**) Curled right hindlimb; (**f**) Head tilt left 30°; (**g**): Head tilt left 60°; (**h**): Head tilt right 30°; (I): Head tilt right 60°; (**j**): Pelvis tilt left 30°; (**k**): Pelvis tilt right 30°; (**l**): Tail tilt left 30°; (M): Tail tilt right 30°; (**n**): Head rotates clockwise; (**o**): Head rotates anti-clockwise

A standardized order of the radiographs (Fig. 4) was utilized for each mouse. The subject was first positioned right in the middle of the plastic pad for the “correct posture” image. After each of the subsequent malpositioning images, the mouse was repositioned into the “correct posture” for further malpositioning. The left forelimb was first pushed towards the body to the limit to yield the “Curled left forelimb” image. Then we performed the similar push maneuver for the right forelimb towards the body to the limit for the “Curled right forelimb” image. The left hindlimb was then pushed towards the root of the tail to the limit for the “Curled left hindlimb” image. Following this, the right hindlimb was then pushed towards the root of the tail to the limit for the “Curled right hindlimb” image.

The mouse was moved by three-finger pinch to adjust its location to be fit for quantification of head tilt. The skull was gently pinched and turned to 30° to the left as measured on the pad scale to produce the “Head tilt left 30°” image. Then the skull was further turned to 60° for the “Head tilt left 60°” image. The skull was then turned 30° to the right for the “Head tilt right 30°” image. The skull was further turned to 60° to the right for the “Head tilt right 60°” image.

For the next step, the mouse was moved by three-finger pinch to adjust its location to be fit for quantification of pelvis tilt. The pelvis was turned 30° to the left by simultaneously rotating the hindlimbs clockwise in terms of the pad scale. This yielded the “Pelvis tilts left 30°” image. The next image entailed turning the pelvis 30° to the right by simultaneously rotating the hindlimbs anti-clockwise. This position yielded the “Pelvis tilt right 30°”.

After moving the mouse by three-finger pinch to adjust its location to be fit for quantification of tail tilting, we gently pushed the tail 30° to the left for the “Tail tilt left 30°” image. The tail was also pushed 30° to the right for the “Tail tilt right 30°” image.

Finally, for the head rotation, we placed the mouse in the middle of the pad again. The skull was pinched and rotated clockwise to the limit for the “Head rotates clockwise” image. The skull was similarly rotated anti-clockwise to the limit for the “Head rotates anti-clockwise”.

### Statistical analysis

The posture change in mouse would result in the change of curve direction. In this part, we regarded the 82 subjects as 82 dependent variables and assigned the curve with apex towards left as negative, and the curve with apex towards right as positive. There were usually three kinds of tests used for the situation that equal variances were not assumed [20]. Dunnett’s T3/Dunnett’s C, Games and Howell and Tamhane’s T2 have the same t ratio and the same df values, but they differ in the *P* value calculation. T2 is more conservative than T3 for large sample sizes while C is more conservative for smaller samples (< 50 per group). The Games-Howell test is suitable for larger sample sizes and is more powerful than C, T2 and T3. Here, One-way ANOVA with Games-Howell tests was used for multiple comparisons with significance level of 0.05.

In order to compare the effect of postures, Glass’s delta was introduced. It is an alternative to Cohen’s d when equal SD is not assumed. It only uses the SD of the control group. Cohen’s d is calculated by the formula: Cohen’s d = |M2 - M1| / SD_pooled_ where M1 represents the mean of control and M2 represents the mean of experimental group. SD_pooled_ is calculated by the formula: SD_pooled_ = √ ((SD12 + SD22) / 2). SD1 and SD2 are the SD of control group and experimental group respectively. A small effect is below 0.2, a small to medium effect is between 0.2–0.45, a medium effect is between 0.45–0.65, a medium to large effect is between 0.65–0.8 and > 0.8 is large effect.

## Results

### Prevalence of scoliosis

For the first batch of 77 mice, we observed a mean Cobb angle measurement of 6.5° ± 5.5°. The standard error of mean was 0.6. Among 77 mice, 23 of them had no curve and 22 of them had a curve that exceeded 10°. The estimated scoliosis prevalence was around 28.6% (22/77) with the “correct posture” image. Relative standard deviation was 84.6% (5.5/6.5) which showed that the curve magnitude had a big variation.

### Measurement error during multiple samplings

To investigate the robustness of curve magnitude, we followed 24 mice for 4 weeks. Weekly X-ray results (Table 1) showed a high mean RSD of 125.3% ± 63.3% suggesting big variations during multiple imaging procedures for mouse scoliosis studies.

**Table 1:** SD, Mean and RSD results of the 24 mice of weekly X-ray experiment

No.	SD	Mean (°)	RSD (%)
1	6.1	9.7	62.7
2	5.2	8.5	60.5
3	4.5	10.2	44.3
4	3.6	5.8	63.0
5	5.5	2.4	223.6
6	3.4	3.7	91.4
7	1.3	0.6	223.6
8	4.4	3.2	138.1
9	3.9	3.4	116.0
10	1.4	0.6	223.5
11	2.6	4.5	56.9
12	4.0	1.8	223.6
13	4.1	4.1	100.4
14	4.5	7.7	58.7
15	3.5	2.6	137.9
16	1.3	0.6	223.6
17	3.2	2.3	140.0
18	3.6	3.7	98.3
19	3.7	2.6	139.1
20	2.8	1.9	143.9
21	3.3	2.2	149.9
22	5.4	4.9	110.5
23	1.4	6.3	21.4
24	3.8	2.3	155.9

SD: standard deviation; RSD: relative standard deviation

### Impact of different postures on the cobb angle measurement

This batch of mice had nearly no curve when in the “correct position” with mean value of − 0.7° ± 5.0°. The effects of “Curled left forelimb”, “Curled right forelimb”, “Curled left hindlimb” and “Curled right hindlimb” were small with − 0.8° ± 4.9°, − 0.6° ± 4.9°, − 0.9° ± 4.8° and − 0.7° ± 5.2°, respectively. “Tail tilt left 30°”, “Tail tilt right 30°”, “Head rotates clockwise” and “Head rotates anti-clockwise” had slightly bigger angles with − 1.0° ± 4.6°, − 1.3° ± 4.6°, − 2.4° ± 4.7° and − 0.7° ± 4.8°, respectively. “Head tilt left 30°”, “Head tilt left 60°”, “Head tilt right 30°”, “Head tilt right 60°”, “Pelvis tilt left 30°” and “Pelvis tilt right 30°” yielded the biggest angles among all 15 postures with 2.2° ± 6.2°, 8.4° ± 8.3°, − 2.1° ± 5.2°, − 8.0° ± 6.6°, 7.1° ± 6.7° and − 8.9° ± 5.9°, respectively (Table 2).

**Table 2:** Mean angle in each posture group and the angle changes with postural changes

Posture		Mean (SD)	Mean difference (i-j)	Std. Error (i-j)	Sig.	95% CI		Minimum	Maximum
Correct posture (i)		−0.7° (5.0)						0°	12.2°
j	Curled left forelimb	−0.8° (4.9)	0.1°	0.8°	1.00	−2.6°	2.8°	0°	11.3°
	Curled right forelimb	−0.6° (4.9)	− 0.2°	0.8°	1.00	−2.8°	2.5°	0°	11.9°
	Curled left hindlimb	−0.9° (4.8)	0.2°	0.8°	1.00	−2.5°	2.8°	0°	11.2°
	Curled right hindlimb	−0.7° (5.2)	0.0°	0.8°	1.00	−2.7°	2.7°	0°	12.2°
	Head tilt left 30°	2.2° (6.2)	−2.9°	0.9°	0.08	−5.9°	0.1	0°	14.8°
	Head tilt left 60°	8.4° (8.3)	−9.2°	1.1°	< 0.001	−12.9°	−5.5°	0°	20.4°
	Head tilt right 30°	−2.1° (5.2)	1.4°	0.8°	0.91	−1.3°	4.1°	0°	11.8°
	Head tilt right 60°	−8.0° (6.6)	7.2°	0.9°	< 0.001	4.1°	10.4°	0°	21.2°
	Pelvis tilt left 30°	7.1° (6.7)	−7.9°	0.9°	< 0.001	−11.1°	−4.7°	0°	18.3°
	Pelvis tilt right 30°	−8.9° (5.9)	8.1°	0.9°	< 0.001	5.2°	11.1°	3.1°	20.8°
	Tail tilt left 30°	−1.0° (4.6)	0.2°	0.8°	1.00	−2.3°	2.8°	0°	10.3°
	Tail tilt right 30°	−1.3° (4.6)	0.6°	0.8°	1.00	−2.0°	3.1°	0°	11.8°
	Head rotates clockwise	−2.4° (4.7)	1.6°	0.8°	0.71	−1.0°	4.2°	0°	14.5°
	Head rotates anti-clockwise	−0.7° (4.8)	−0.0°	0.8°	1.00	−2.6°	2.6°	0°	11.6°

*SD* standard deviation

Mean differences between the “correct posture” and other 14 postures followed the similar distributional pattern. All postures related to tilting of the head or pelvis except for “Head tilt 30°” showed statistically significant difference compared to “correct posture” (Table 2). The effect size of “Curled left forelimb” (0.02), “Curled right forelimb” (0.02), “Curled left hindlimb” (0.04) and “Curled right hindlimb” (0.00) were small. Similarly the effect sizes for “Tail tilt left 30°” (0.05), “Tail tilt right 30°” (0.11) and “Head rotates anti-clockwise” (0.00) groups were small. “Head rotates clockwise” showed a small to medium effect size of 0.32. Yet, “Head tilt left 60°” (1.83), “Head tilt right 60°” (1.44), “Pelvis tilt left 30°” (1.57) and “Pelvis tilt right 30°” (1.62) had very big Glass’s delta values indicating a large effect of these postures on angle measurement (Table 3).

**Table 3:** Glass’s delta for each posture

	Glass’s delta
Curled left forelimb	0.02
Curled right forelimb	0.03
Curled left hindlimb	0.04
Curled right hindlimb	0.00
Head tilt left 30°	0.58
Head tilt left 60°	1.83
Head tilt right 30°	0.28
Head tilt right 60°	1.44
Pelvis tilt left 30°	1.57
Pelvis tilt right 30°	1.62
Tail tilt left 30°	0.05
Tail tilt right 30°	0.11
Head rotates clockwise	0.32
Head rotates anti-clockwise	0.00

## Discussion

Scoliosis is not necessarily a deadly disease, but it affects the patients’ cosmesis, mobility, pain and quality of life [21, 22]. The diagnosis and management decision of scoliosis relies on the Cobb angle measurement and thus these measurements must be accurate and consistent. The measurement error for human subjects has already been exemplified from various aspects [5, 8, 23–25]. However the effects of mouse positioning on angle measurement is unclear. The mouse is a commonly used animal model to study scoliosis as it suits both mechanical and genetic testing [26]. With this study, we identified up to 28.6% of wild-type mice with scoliosis despite a “correct posture” image. There are large variations with multiple imaging of a single mouse. Positioning is important as we found head and pelvic tilting to greatly affect the Cobb angle measurement.

To fulfill the purpose of investigating the measurement error during radiograph imaging, we utilized three distinct experiments. The first study entails an overview of the prevalence of scoliosis in the “normal” mouse population through imaging 77 C57BL/6 J mice in the supposedly “correct posture”. Using the 10° diagnostic criteria for humans, we identified up to 28.6% of the mice to have scoliosis. Furthermore, up to 70.1% has some curvature despite most not fulfilling the 10° diagnostic threshold. This suggests that quadrupedal animals may have high prevalence of spinal curvatures. This has implications on the effectiveness of the mechanical or genetic insult used to produce a spinal curvature. The severity of the curvature or the number of overall animals affected becomes a more valid measure than whether a curvature exists or not. Furthermore, future animal studies on scoliosis will need to report on a standardized method of radiographic measurement for data validity and accuracy. The presence of any scoliosis may be a result of normal spinal positional variance.

It is also important to note the potential effects of anesthesia on curve magnitude. Similar to human studies where effects of posture may lead to changes in spinal alignment and severity [7, 27], variations in muscle tone may lead to measurement variability. The mean RSD was 125.3% which indicated a big variation and this serves as a guide to the degree of variability that exists in every radiographic measurement. The likely reason for the large variation is the measurements done on immature mice with weekly intervals. The spinal deformity may have progressed as a result of growth as these immature mice have yet to reach adulthood. As with human studies, curve pattern changes through growth may occur [28, 29] and thus studies should standardize the age of subjects to avoid this influence.

From this study, we have identified the “correct posture” which should be performed in all mouse studies assessing the spinal curvature. Researchers should be cautious when interpreting radiographs of animals with malpositioning especially of the head and pelvis as we have showed differences of up to 8–9°. Based on the 5° measurement error in Cobb angle as reported in human studies [8], our findings have surpassed the clinical significance threshold. This error may lead to misclassification into false positive results by placing a normal subject into the scoliosis group or false negative results by placing a scoliotic subject into the normal group. As the head and pelvis are closely linked to the axial skeleton, malpositioning of these two body parts have a striking effect on the curvature of the spine. Conversely, altering the appendicular skeleton by curling of the limbs and tilting of the tail have minimal effects on the spinal curvature. The forelimbs and hindlimbs are left-right symmetrical and are perpendicular to the spine. They are also binary components in the mouse and less movement is contributed to the femur and humerus. The glenoid cavity also provides great buffer to the movement of forelimbs [30] that the balance of the scapulae is seldom interrupted. The components of the sacrum and ilium also provides mechanical support to the spino-pelvic complex which helps resist pelvic tilting as a result of femur or tail movement. Our findings for head tilt show clinically significant variations in the Cobb angle with 60° tilting rather than 30° tilting. The head tilt effect is not as significant as the pelvic tilt in which 30° already produces significant changes in angle measurement. The cervical spine acts like a buffer to head movement and so the effect only becomes prominent with large distortions. As safe rotation of the head only allows a small degree of motion as compared to head tilting, the effects on angle variation is minimal and thus should not be a significant factor to consider when positioning the mice for radiographs. It is also important to note rotational changes. If the forelimb is pressed under the chest for example, as compared to against the chest as we have done in this study, the chest would be lifted by the forelimb. As a result, the head will rotate towards the ipsilateral side and the natural kyphosis of the spine may introduce a false scoliosis into view.

One important limitation to this study is the use of wildtype mice. In mechanically and genetically modified mice, we may not see similar effects with malpositioning. The prevalence of scoliosis in those mice may also differ. Hence, there may be a selection bias in the population. The use of 10° as the cut-off was to follow the criteria in humans. However, humans are bipedal while mice are quadrupedal and thus this cut-off may not be appropriate. Nevertheless, our study aim is to suggest the correct posture for radiographs rather than diagnosis of scoliosis. In altered neuromuscular states, the variance may be even larger so that forelimb and hindlimb malpositioning may cause significant curve pattern changes. It is thus recommended to provide the “correct posture” for all cases and be cautious to have the limbs in a symmetrical posture as well. There is also an assumption that the mouse is left-right symmetrical and that we should place the “correct posture” with the two sides symmetrical. Hence, our “correct posture” is the recommended position for this batch of wild-type mice with symmetrical measurements. It needs to be verified as “correct” also for mice with asymmetry whereby this posture may correct the spine and mask any subtle deformity. Scoliosis is also a 3-dimensional deformity and appreciation of the sagittal and axial planes are important. This requires further standardization and study. It should also be studied in mice with genetic predisposition to spinal deformity. Furthermore, it is also important to note that the radiographs were done with the x-ray tube centralized to the platform in a neutral position. The effects of tilting the x-ray tube on the spine may require further exploration.

## Conclusion

From this radiographic animal study, we detected large variances in curve magnitude within the mouse population. The standard recommended posture for mouse imaging entails positioning the snout, interorbital space, neck and whole spine in one line, and with the limbs placed symmetrical to the trunk, whilst avoiding stretching the body of the mouse. Curling of the limbs, tilting of the tail and rotation of the head had minor effects on the curve magnitude. The greatest effects are contributed by head and pelvic tilting. Researchers must be vigilant in mouse positioning during radiographs to avoid any disturbance to the spine, leading to invalid imaging data. Only with standardization of the posture for radiographs can we cross-compare results and provide justified diagnoses of spinal deformities. Head or pelvic tilting may lead to false representation of the curve or exaggerate the spinal deformity. Future studies should identify variations within mechanically and genetically altered mice as well as the effects of posture on other imaging modalities such as CT and MRI.

## Data Availability

The data is kept by the corresponding author and is available upon request.

## Abbreviations

ANOVA: analysis of variance
RSD: Relative standard deviation
SD: standard deviation
